# Gender differences in the prevalence and determinants of tobacco use among school-aged adolescents (11 – 17 years) in Sudan and South Sudan

**DOI:** 10.11604/pamj.2014.18.118.3202

**Published:** 2014-06-05

**Authors:** Dominic Odwa Atari

**Affiliations:** 1 Department of Geography, Nipissing University, North Bay, Ontario, Canada

**Keywords:** Adolescents, prevalence, cigarette, tobacco, Sudan, South Sudan

## Abstract

**Introduction:**

Tobacco use is one of the leading and preventable causes of global morbidities and premature mortalities. The study explores gender differences in the prevalence and determinants of tobacco use among school-aged adolescents (11-17years) in Sudan and South Sudan.

**Methods:**

The study utilized the national Global Youth Tobacco Survey (GYTS) data collected in 2005 for Sudan (4,277 unweighted; 131,631 weighted). Univariate and bivariate analyses were conducted to examine the associations between the dependent (tobacco use status) and independent variables. Logistic regression analyses were performed to identify the key factors which influence tobacco consumption among adolescents in the 2 Sudans for ever cigarette users, current cigarette users, and users of noncigarette tobacco products.

**Results:**

There were significant gender differences in the prevalence of ever cigarette users (21.8%; male=13.1%, female=6.5%, p<0.05) and current cigarette users (6.9%; male=4.9%, female = 1.3%, p<0.05) but not among users of noncigarette tobacco products (14.7%; male=6.8%, female=6.1%). Adolescent tobacco use was significantly associated with availability of monthly income or allowance, exposure to tobacco industry promotions, and tobacco-use behavior of familial relations. Knowledge about the harmful effects of secondhand smoke was related with decreased likelihood of tobacco use.

**Conclusion:**

School programs that focus on health messages alone may not work for the adolescent population. Legislations that ban all types of tobacco advertisements, promotions, and sponsorships among adolescents are needed in the 2 countries.

## Introduction

Tobacco use is one of the leading and preventable causes of global morbidities and premature mortalities including cancer and cardiovascular diseases [1, 2]. Currently, there are more than 1 billion smokers worldwide [2] of which more than 150 million are children [3] and more than half will die prematurely [1, 4, 5]. In the year 2000 alone, about one million deaths were attributed to cigarette smoking worldwide with most increase noted in low-and-medium income countries (LMICs) [6] where more than 80% of the global tobacco users reside [2, 7]. By 2030, about 70% of the expected 10 million tobacco-related deaths will occur in the developing world, if anti-tobacco programs are not developed and enforced [8]. Unfortunately, the epidemic increase of tobacco consumption in the developing world will inflict major public health impacts [9, 10] and affect economic growth and development particularly in very poor countries like (north) Sudan and South Sudan which already face considerable challenges of fighting poverty and infectious diseases.

In 2005, the global prevalence of cigarette smoking was far higher among men (41.9%) than women (8.9%) [11] but the trends are changing with more and more women smoking. The prevalence of current cigarette smoking among boys and girls in Africa is 13% and 5.8%, respectively [12], and the annual cigarette consumption rates are on the rise for both men and women [13]. According to the Centers of Disease Control and Prevention, Kenya has a current national smoking prevalence of about 8.2% (male=11.2%, female=5.2%) among school-aged adolescents (13-15 years). Uganda has a current smoking prevalence of 5.5% (male=6.6%, female=4.0%) while Ethiopia has a prevalence of 1.9% with 2.5% and 0.9% among young men and women, respectively [14]. The 2001 Sudan national Global Youth Tobacco Surveys (GYTS) showed a prevalence of 17.1% (male=25.6%, female=6.1%) for ever cigarette users, 6.1% (male=10.8%, female=1.9%) for current cigarette users, and 13.5% (male=17.2%, female=10.4%) for users of noncigarette tobacco products [14]. With the exception of El-Amin et al. [15], who explored the role of parents, friends, and teachers in adolescent cigarette smoking and tombak dipping in Sudan, there have been no recent studies in Sudan or South Sudan that explore the factors which influence tobacco use in the region. Hence this study aims at examining gender differences in the prevalence and determinants of tobacco use among school-aged adolescents using the 2005 national Sudan GYTS - the only current dataset available for the 2 countries.

Policy Context: Description of the 2 Sudans

After several years of conflict, the political and economic landscapes of Sudan have significantly changed. The war between the Government of the Republic of Sudan and the Sudan People′s Liberation Movement/Army ended after the signing of the Comprehensive Peace Agreement (CPA) in 2005. The CPA ended after 6 years interim period with a referendum for independence which resulted in the separation of South Sudan from Sudan in 2011 but did not resolve the conflicts in the Darfur, Blue Nile, and Southern Kordofan regions, and the disputed border region of Abyei. Before the secession, Sudan signed the World Health Organization Framework Convention on Tobacco Control (FCTC) in 2004, ratified and operationalized it in 2005 and 2006, respectively. However, South Sudan did not inherit the signed FCTC [16]. There has been some interest from the ministry of health to enact tobacco control legislations to prevent the excessive consumption of tobacco and other drugs in South Sudan [17] but its legislators are yet to show some interest. Currently, there are no legislations protecting children from obtaining tobacco products in the 2 countries. In addition, there are no regulations banning some or all types of tobacco advertisement, promotion and sponsorship. However, the onset of tobacco use occurs primarily in early adolescence [18] making it an important public health challenge [13], especially in the 2 Sudans where the majority (41.7%) of the population is under 15 years old [19, 20]. Regrettably, many of the young men and women will become daily cigarette users before they could comprehend the adverse health impacts of their behaviors [1]. The study will add to the sparse literature on tobacco usage among boys and girls in the region and other LMICs and enhance the capacity of the 2 Sudans to design, implement, and evaluate their respective national tobacco action plans.

## Methods

### Data collection

The study was based on the standardized GYTS questionnaire modified for Sudan. The GYTS is a surveillance program developed by the World Health Organization (WHO), the U.S. Centers for Disease Control and Prevention (U.S. CDC), and the Canadian Public Health Association (CPHA) to address the limited country and regional specific data availability [21]. The GYTS is a school-based survey which collects information on the use of cigarette and noncigarette tobacco products on five major determinants including access/availability and price of tobacco products, exposure to second hand smoke (SHS), tobacco-use cessation, role of media and advertising, and school curriculum among school-aged adolescents [22].

The study data was collected in 2005 before South Sudan seceded from Sudan. In total, a sample of 4,277 unweighted (131, 631 weighted) school-going adolescents aged 11-17 years were surveyed. The GYTS dataset is publicly available from the CDC and detailed description of the sampling method and data collection procedures have been published elsewhere [12, 22, 23]. Briefly, the GYTS was a two-step cluster sampling design to gather data about tobacco use in school-going adolescents. At the first stage, clusters of schools were collected with probability proportional to their enrollment sizes. During the second stage, classrooms were randomly selected in each school cluster, and all students in the classrooms were eligible to participate in the survey. The school, class, and student response rates were 92%, 100%, and 93.2%, respectively, with an overall response rate of 85.7% [14].

To estimate the prevalence of tobacco use among adolescents in Sudan, the data were weighted to adjust for design effect (selection of school and class levels), nonresponses (school, class, and student levels), and post-stratification of the sample population relative to the grade and sex distribution in the population. This method of weighing measures has been previously explained elsewhere [24]. The weighting factor is given by the formula: W=W1 * W2 * f1 * f2 * f3 * f4 Where W1 is the inverse of the probability of selecting a school; W2 is the inverse of the probability of selecting a classroom within a school; f1 is a school-level nonresponse adjustment factor calculated by school size category (small, medium, large); f2 is a class-level nonresponse adjustment factor calculated for each school; f3 is a student-level nonresponse adjustment factor calculated by class; and f4 is a post-stratification adjustment factor calculated by sex and grade. The questionnaires were distributed to students attending classes and completed during class time with the supervision of a specially trained research staff. The approach made sure no teacher was present during the survey which lasted about 45 minutes.

### Measurements

The outcome variable was measured as tobacco-use status of respondents based on three questions: (a) “Have you ever tried or experimented with cigarette smoking, even one or two puffs-” (*Ever Tobacco Users*); (b) “During the past 30 days (one month), on how many days did you smoke cigarettes-” (*Current Tobacco Users*); (c) “During the past 30 days (one month), have you ever used any form of tobacco products other than cigarettes-” (Users of noncigarette Tobacco Product). In general, the outcome variables were dichotomized into 1 if the person is an ever cigarette user or user of other tobacco products and 0 if they were not. The current cigarette users were recoded as 0 if the person did not smoke in any day in the past 30 days and 1 if they smoked in the past 30 days. The independent variables included age, sex, knowledge of SHS, tobacco-use behavior of familial relations, media and advertising, tobacco industry promotion, school curriculum, and attitude toward smoking bans in public places.

### Data Analysis

Bivariate and multivariate analyses were conducted to determine the relationship between the dependent variables (tobacco-use status) and independent variables. Unweighted frequencies and weighted percentages were reported. Differences in tobacco-use status across categories were assessed using a t test for continuous variables and a χ^2^ test for categorical variables [25]. Logistic regression analyses were used to determine the associations between the dependent and independent variables. Individual logistic regression analyses were conducted for ever cigarette users, current cigarette users, and users of noncigarette tobacco products. In addition, stratified analyses were performed based on gender to delineate the determinants associated with tobacco use among school-aged adolescent boys and girls in the 2 Sudans. Proc SURVEYLOGISTIC was used from SAS to calculate the adjusted odd ratios (*OR*) along with respective 95% confidence intervals (*CI*). All data analyses were performed using SAS for Windows, Version 9.2 [26].

## Results

Table 1 shows the unweighted frequencies and weighted percentages of adolescents- tobacco use in the 2 Sudans. Of the total sample population (unweighted = 4,277, weighted = 131,631), 21.8% were ever cigarette users (13.1% males and 6.5% females), 6.9% were current cigarette users (4.9% males and 1.3 females), and 14.7% were users of noncigarette tobacco products (6.8% males and 6.1% females). The -2 test analyses show that males were significantly more likely to be ever and current cigarette users (*p* < .05) than their female counterparts. There were no significant gender differences between users of noncigarette tobacco products. However, the analyses indicate that the prevalence of tobacco use was highest among 14 and 15 years old adolescents.

**Table 1:** Unweighted frequencies and weighted percentages of adolescents’ tobacco use in the 2 Sudans, 2005

Characteristics	Total (4,277)	Ever Cigarette Users 21.8% (1,022)	Current Cigarette Users 6.9% (320)	Noncigarette Users 14.7% (513)
**Age**				
11 years old or younger	4.9 (125)	1.7 (44)	0.4 (13)	2.0 (47)
12 years	7.1 (181)	1.1 (32)	0.3 (8)	1.5 (35)
13 years	20.6 (601)	3.1 (112)	1.2 (39)	1.9 (55)
14 years	26.6 (1,090)	5.0 (238)	1.3 (55)	2.6 (85)
15 years	22.7 (1,140)	4.7 (272)	1.5 (84)	2.3 (105)
16 years	12.5 (615)	2.9 (171)	1.0 (61)	2.0 (90)
17 years old or older	5.6 (294)	1.9 (97)	0.6 (39)	0.8 (41)
(Missing)	(231)	1.4 (56)	0.6 (21)	1.7 (55)
**Sex**				
Male	48.3 (2,270)	13.1 (719)	4.9 (251)	6.8 (269)
Female	51.7 (1,740)	6.5 (222)	1.3 (46)	6.1 (185)
Missing	(267)	2.3 (81)	0.8 (23)	1.8 (59)
**Education**				
Basic 8 class	47.1 (902)	4.4 (168)	1.4 (52)	4.0 (149)
Secondary 1st class	29.1 (1,595)	9.9 (380)	2.9 (111)	4.3 (162)
Secondary 2nd class	23.8 (1,461)	10.3 (392)	3.4 (132)	4.0 (140)
**Perception about tobacco use**				
Boys: smokers have less friends	54.1 (2,270)	10.5 (419)	3.0 (120)	5.2 (204)
Girls: smokers have less friends	63.8 (2,708)	11.8 (578)	3.4 (162)	6.8 (253)
Boys: smokers less attractive	58.9 (2,518)	12.5 (659)	3.4 (184)	5.9 (284)
Girls: smokers less attractive	70.7 (2,985)	13.9 (498)	4.0 (137)	8.0 (232)
**Knowledge about Health effects of tobacco products**				
Smoking is harmful	83.6 (3,695)	18.7 (909)	6.0 (287)	10.6 (401)
SHS is harmful	78.3 (3,467)	17.8 (864)	5.5 (265)	9.4 (367)
**Tobacco use behavior of familial relations**				
Live in a home with one or more smokers	18.6 (832)	9.9 (439)	3.6 (170)	4.7 (174)
Around others who smoke outside home	41.2 (1,897)	12.9 (625)	4.8 (231)	5.5 (212)
Have one or more parents who smoke	16.6 (719)	5.2 (246)	1.9 (87)	2.7 (102)
Have some or all friends who smoke	39.3 (1,753)	11.6 (562)	4.5 (220)	5.9 (219)
**Media and advertising**				
Seen cigarette billboard in past 30 days	53.4 (2,175)	13.6 (604)	4.4 (209)	9.6 (314)
Seen cigarette ad in newspaper or magazine in past 30 days	51.1 (1,982)	12.4 (514)	4.3 (175)	9.0 (288)
Tobacco industry promotion				
Owned object with cigarette brand logo	19.5 (771)	5.7 (243)	2.2 (95)	4.5 (146)
Offered free cigarette by tobacco company rep	13.3 (465)	4.3 (157)	1.7 (67)	5.0 (140)
**School Curriculum**				
Taught about the dangers of smoking in past year	33.0 (1,261)	13.9 (686)	4.6 (220)	8.3 (304)
Discussed why people at young age should not smoke	23.3 (827)	15.5 (763)	5.0 (242)	8.9 (334)
Taught about the effects of smoking	32.1 (1,147)	14.6 (711)	4.8 (221)	7.7 (282)
**Attitude toward smoking ban**				
Favor	82.5 (3,535)	4.4 (168)	1.8 (72)	2.7 (83)

The logistic regression analyses (Table 2) show the key determinants of tobacco-use status among school-aged adolescents (11 - 17 years). The adjusted estimates show that age had a significant influence in ever cigarette tobacco use. The model reveals that males were 2.73 and 4.00 times more likely to be ever cigarette users (OR=2.73, 95% CI (2.12 - 3.50)), and current cigarette users (OR=4.00, 95% CI (2.39 - 6.70)), respectively, than their female counterparts. Educational level indicates significant influence on the use of noncigarette tobacco products. Adolescents who were in basic 8 class were 36% more likely (OR=1.36, 95% CI (0.85 - 2.16)) but those who were in secondary one level were 15% less likely (OR=0.85, 95% CI (0.60 - 1.19)) to be users of noncigarette tobacco products compared to their counterparts who were in secondary 2 class.

**Table 2:** Smoking Status among Adolescents in the 2 Sudans, 2005

		Ever Cigarette User	Current Cigarette User	User of Noncigarette Tobacco Product
		Adjusted OR (95% CI)	Adjusted OR (95% CI)	Adjusted OR (95% CI)
**SocioDemographic Variables**				
Age (Continuous)		1.12(1.00-1.25)*	1.14(0.97-1.35)	1.05(0.92-1.20)
Education (Secondary 2 level)	Primary 8 level	1.09(0.76-1.57)	1.22(0.68-2.20)	1.36(0.85-2.16)*
	Secondary 1 level	1.01(0.80-1.28)	1.06(0.72-1.54)	0.85(0.60-1.19)**
Male		2.73(2.12-3.50)***	4.00(2.39-6.70)***	1.23(0.90-1.69)
Pocket money (Continuous)		1.12(1.02-1.23)**	1.31(1.12-1.53)***	1.07(0.93-1.23)
**Knowledge about secondhand smoke (SHS)**				
SHS is Harmful		0.48(0.31-0.75)***	0.53(0.25-1.12)*	0.56(0.35-0.89)**
**Tobacco-use behavior of familial relations**				
Live in home with one or more smoking persons		1.65(1.26-2.18)***	1.58(1.00-2.49)*	1.20(0.84-1.71)
Around others who smoke outside home		2.21(1.69-2.88)***	3.02(1.68-5.44)***	1.08(0.76-1.53)
Have one or more parents who smoke		1.55(1.14-2.10)***	1.12(0.66-1.89)	1.17(0.78-1.76)
Have most or all friends who smoke		1.54(1.22-1.93)***	2.55(1.64-3.94)***	0.99(0.72-1.36)
**Media and Advertising**				
Seen cigarette billboard in past 30 days		1.23(0.94-1.60)	0.96(0.59-1.56)	1.18(0.80-1.75)
Seen cigarette ad in newspaper or magazine in the past 30 days		0.94(0.72-1.23)	1.34(0.86-2.10)	0.99(0.67-1.47)
**Tobacco industry promotion**				
Owned object with cigarette brand logo		1.36(1.04-1.79)**	1.76(1.10-2.82)**	1.78(1.24-2.56)***
Offered free cigarette by tobacco company representative		1.67(1.12-2.49)**	2.49(1.38-4.51)***	4.39(2.92-6.60)***
**School curriculum**				
Taught about dangers of smoking in past year		1.04(0.78-1.38)	0.81(0.48-1.37)	1.16(0.82-1.63)
Discussed why people at young age should not smoke		0.92(0.66-1.28)	0.86(0.47-1.58)	0.90(0.59-1.39)
Taught about effects of smoking		0.87(0.64-1.18)	0.93(0.53-1.62)	1.23(0.82-1.84)
**Attitude toward smoking ban**				
Support ban		0.75 (0.53-1.08)	0.43(0.26-0.72)***	1.46(0.91-2.33)

Monthly income or allowance appears to be an important risk factor for using tobacco products among adolescents. The risk of becoming an ever cigarette user and current cigarette user increased by 12% and 31%, respectively, with a 40,000 Sudanese pounds (∼9 US dollars in 2005) unit increase in monthly allowances. Adolescents who think that SHS is harmful were less likely to be ever cigarette users (OR=0.48, 95% CI (0.31 - 0.75)), current cigarette users (OR=0.53, 95% CI (0.25 - 1.12)), and users of noncigarette tobacco products (OR=0.56, 95% CI (0.35 - 0.89)) than their counterparts who believe otherwise.

When tobacco-use behavior of familial relations were explored, living in a home with one or more smoking persons, having one or more parents who smoke, and having most or all friends smoking showed significant associations with increased probability of tobacco use. In particular, living with one or more smoking persons increased the likelihood of being an ever cigarette user (OR=1.65, 95% CI (1.26 - 2.18)) and current cigarette user (OR=1.58, 95% CI (1.00 - 2.49)). On the other hand, living around others who smoke increased the likelihood of being an ever cigarette smoker by more than 2 times (OR=2.21, 95% CI (1.69 - 2.88)) and current cigarette user by more than 3 times (OR= 3.02, 95% CI (1.68 - 5.44)). Having one or more smoking parents increased the likelihood of being an ever cigarette user by 1.55 times (OR=1.55, 95% CI (1.14 - 2.10)) among school-aged adolescents. Study subjects who had most or all their friends smoking were 1.54 times more likely to be ever cigarette users (OR=1.54, 95% CI (1.22 - 1.93)) but almost 3 times more likely to be current cigarette users (OR=2.55, 95% CI (1.64 - 3.94)) compared to those who do not have such peers.

Media and advertising related variables show no significant influence in tobacco use among adolescents in the 2 Sudans. However, the results indicate that tobacco industry promotion significantly increased the likelihood of adolescents in becoming cigarette users. For instance, the adjusted estimates reveal that owning objects with a cigarette brand logo significantly increased the likelihood of being an ever cigarette user by 1.36 times (OR=1.36, 95% CI (1.04 - 1.79)), current cigarette user by 1.76 times (OR=1.76, 95% CI (1.10 - 2.82)), and user of noncigarette tobacco products by 1.78 times (OR=1.78, 95% CI (1.24 - 2.56)). When offered a free cigarette by a tobacco company representatives, adolescents were between 1.67 and 4.39 times more likely to be ever cigarette users (OR= 1.67, 95% CI (1.12 - 2.49)), current cigarette users (OR=2.49, 95% CI (1.38 - 4.51)), and users of noncigarette tobacco products (OR=4.39, 95% CI (2.92 - 6.60)), respectively. Adolescents who support cigarette ban in public places were 57% less likely to be current cigarette users (OR=0.43, 95% CI (0.26 - 0.72) compared to their counterparts who support no ban.

When the stratified analyses based on gender were conducted, the same factors in the general model were identified to be associated with tobacco use among male and female adolescents in the 2 Sudans but with minor variations in the strength of association and level of significance (Table 3). In the male specific model, the risk of becoming a current cigarette user increased by 28% with a 40,000 Sudanese Pounds unit increase in the monthly income or allowance. However, the risk of being an ever cigarette user (OR=1.16, 95% CI (1.00 - 1.34)), current cigarette user (OR=1.34, 95% CI (1.1.03 - 1.75)) and user of noncigarette tobacco products (OR=1.18, 95% CI (0.97 - 1.44)) for the female adolescents increased by 16%, 34%, and 18%, respectively, with the same unit increase of monthly income or allowance. A major difference occurred in the media and advertising variables where seeing cigarette advertisements on billboard significantly increased the likelihood for male adolescents to be users of noncigarette tobacco products by 1.69 times (OR=1.69, 95% CI (1.05 - 2.74)).

**Table 3:** Gender differences in Smoking Status among Adolescents in the 2 Sudans

	Male				Female		
	Ever Cigarette User		Current Cigarette User	User of Noncigarette Tobacco Product	Ever Cigarette User	Current Cigarette User	User of Noncigarette Tobacco Product
	Adjusted OR (95% CI)		Adjusted OR (95% CI)	Adjusted OR (95% CI)	Adjusted OR (95% CI)	Adjusted OR (95% CI)	Adjusted OR (95% CI)
**SocioDemographic Variables**							
Age (Continuous)	1.10(0.96-1.26)		1.24(1.03-1.50)**	1.05(0.88-1.24)	1.15(0.93-1.42)	0.75(0.50-1.13)	1.07(0.85-1.34)
Education (Secondary 2 level)	Primary 8 level	0.84(0.54-1.32)	1.25(0.63-2.48)	1.12(0.59-2.14)	1.85(0.95-3.60)	1.36(0.45-4.11)	1.62(0.77-3.40)**
	Secondary 1 level	0.91(0.70-1.19)	0.91(0.61-1.34)	0.96(0.65-1.44)	1.38(0.83-2.30)	2.34(0.68-7.99)	0.65(0.34-1.25)**
Pocket money (Continuous)	1.10(0.97-1.24)		1.28(1.06-1.54)**	1.01(0.83-1.23)	1.16(1.00-1.34)*	1.34(1.03-1.75)**	1.18(0.97-1.44)*
**Knowledge about secondhand smoke (SHS)**							
SHS is Harmful	0.54(0.31-0.94)**		0.58(0.24-1.39)	0.68(0.37-1.26)	0.49(0.25-0.97)**	0.58(0.21-1.64)	0.51(0.25-1.03)*
Tobacco-use behavior of familial relations							
Live in home with one or more smoking persons	1.95(1.41-2.69)***		1.76(1.06-2.93)**	1.20(0.78-1.84)	1.32(0.77-2.27)	1.24(0.43-3.58)	1.20(0.64-2.25)
Around others who smoke outside home	1.89(1.38-2.6)***		3.16(1.58-6.31)***	0.87(0.57-1.34)	2.71(1.68-4.36)***	3.07(0.85-11.04)*	1.27(0.73-2.22)
Have one or more parents who smoke	1.34(0.92-1.95)		1.26(0.70-2.28)	1.52(0.94-2.48)*	2.08(1.21-3.58)***	0.73(0.24-2.26)	0.86(0.39-1.87)
Have most or all friends who smoke	1.55(1.17-2.04)***		2.19(1.37-3.49)***	1.36(0.90-2.07)	1.53(1.01-2.30)**	4.49(1.65-12.19)***	0.63(0.37-1.08)*
**Media and Advertising**							
Seen cigarette billboard in past 30 days	1.25(0.91-1.71)		0.89(0.53-1.51)	1.69(1.05-2.74)**	1.21(0.76-1.93)	1.66(0.56-4.98)	0.82(0.44-1.54)
Seen cigarette ad in newspaper or magazine in the past 30 days	0.98(0.71-1.37)		1.35(0.82-2.21)	0.90(0.56-1.43)	0.86(0.53-1.38)	1.18(0.43-3.25)	1.13(0.59-2.14)
**Tobacco industry promotion**							
Owned object with cigarette brand logo	1.59(1.14-2.20)***		1.88(1.12-3.16)**	1.84(1.16-2.93)**	1.10(0.65-1.83)	1.30(0.36-4.68)	1.93(1.11-3.36)**
Offered free cigarette by tobacco company representative	1.45(0.88-2.37)		2.32(1.18-4.57)**	4.57(2.66-7.83)***	2.02(1.08-3.77)**	4.92(1.70-14.23)***	4.60(2.44-8.68)***
**School curriculum**							
Taught about dangers of smoking in past year	0.97(0.69-1.35)		0.70(0.39-1.24)	1.18(0.77-1.83)	1.22(0.70-2.12)	1.84(0.50-6.76)	1.06(0.59-1.90)
Discussed why people at young age should not smoke	1.05(0.70-1.57)		0.84(0.43-1.63)	0.99(0.57-1.72)	0.74(0.40-1.37)	0.99(0.28-3.46)	0.89(0.44-1.79)
Taught about effects of smoking	0.94(0.64-1.37)		1.09(0.60-1.97)	1.04(0.63-1.71)	0.66(0.36-1.19)	0.38(0.09-1.58)	1.49(0.75-2.95)
**Attitude toward smoking ban**							
Support ban	1.02(0.65-1.61)		0.53(0.29-0.97)**	1.63(0.88-3.02)	0.50(0.30-0.83)***	0.19(0.07-0.53)***	1.53(0.75-3.11)

Male respondents who owned objects with cigarette brand logo were 1.59 times more likely to be ever cigarette users (OR=1.59, 95% CI (1.14-2.20)), 1.88 times to be current cigarette users (OR=1.84, 95% CI (1.12 -3.16)), and 1.84 times to be users of noncigarette tobacco products (OR=1.84, 95% CI (1.16 - 2.93)). Similarly, females were almost 2 times more likely to be users of noncigarette tobacco products (OR=1.93, 95% CI (1.11 - 3.36)) if they had objects with cigarette brand logos. When offered free cigarette by tobacco company representative, males were 2.32 times (OR=2.32, 95% CI (1.18 - 4.57)) and 4.57 times (OR=4.75, 95% CI (2.66 - 7.83)) more likely to be current tobacco users and users of noncigarette tobacco products but females were between 2.02 and 4.92 times more likely to be ever cigarette users, current cigarette users, and users of noncigarette tobacco products (Table 2).

Supporting smoking ban in public places had a greater influence on female respondents than their male counterparts. For instance, males were only 47% less likely to be current cigarette users (OR=0.53, 95% CI (0.29-0.97)) but females were 50% and 81% less likely to be current cigarette users (OR=0.50, 95% CI (0.30 - 0.83)) and users of noncigarette products (OR=0.19, 95% CI (0.07 - 0.53)), respectively, if they supported a ban. In general, tobacco industry promotion and tobacco-use behavior of familial relations significantly increased the likelihood of using tobacco products, but the knowledge that SHS is harmful decreased tobacco use among school-going adolescents in the 2 Sudans.

## Discussion

The study explored gender differences in the prevalence and determinants of tobacco use among school-aged adolescents in (north) Sudan and South Sudan using the 2005 Sudan national GYTS. The results show significant gender differences in the prevalence of ever cigarette users and current cigarette users but not among users of noncigarette tobacco products (Table 1). These findings are similar to other studies which reported a wide gender gap in smoking habits in East Africa [13, 14] denoting a traditional norm of tobacco consumption in the region [12, 27, 28]. However, the results are different from Mamudu et al.‘s who found a narrow gap in tobacco use between males and females in Ghana [29].

When compared, the prevalence of current cigarette users among adolescents in the 2 Sudans (6.9%, Table 1) is lower than that found in Kenya (8.2%), but higher than that found in Uganda (5.5%) and Ethiopia - Addis Ababa (1.9%) [14]. This could partly be because of the different age ranges used. This study used a wider age range (11-17 years) compared to a narrow age range (13-15 years) used for these countries [14]. The prevalence of noncigarette tobacco product users found in this study (14.7%; male=6.8; female=6.1) is similar to that in Uganda (13.9%) but not Kenya (10.1%; male=11.2, female=5.2%) and Ethiopia - Addis Ababa (6.6%; male=8.4%, female=4.4%) [14].

The narrow gap between users of noncigarette tobacco products in this study raises a concern of whether female adolescents perceive noncigarette tobacco products to be less toxic or addictive and more socially acceptable. Anecdotal evidence shows that both male and female adolescents in the region are publicly smoking water pipes also known as shisha. The use of flavored cigarette products marketed as noncigarette products in sisha could be a “gateway” to cigarette smoking and the industry effort to lure young people to switch to such product is high [31]. In addition, studies have shown that girls may develop signs of tobacco dependency at significantly faster rate than boys [32, 33].

The fact that education and monthly income or allowance have significant influence on adolescent smoking habits suggest that socioeconomic factors are important risk factors for smoking in the 2 countries [34, 35]. These findings are similar to other studies in South Africa which reported significant associations between tobacco use and improved socioeconomic statuses [36, 37].

In general, tobacco use has become a “pediatric disease” because most cigarette users begin when they are adolescents [38, 39]. In this study, the prevalence of ever cigarette users, current cigarette users, and users of noncigarette tobacco products was highest among adolescents who were between 14 and 15 years old. When compared, the prevalence of ever cigarette users among adolescents in this study (21.8%) was greater than the prevalence measured in the previous 2001 Sudan national GYTS (17.1%) [14]. The increasing trend in ever cigarette users is a health concern that urgently needs to be addressed specially in a region where the majority of the population is under 15 years of age [19, 20]. It is therefore the responsibility of the public health workers, educators, and the governments in the 2 countries to enact comprehensive anti-tobacco policies and public health education and prevention efforts to reduce the potential health, economic, social and political impacts of high tobacco consumption in the region.

The fact that tobacco-use behavior among school-aged adolescents in the 2 Sudans was associated with tobacco industry promotions affirms earlier studies which show industrial promotion as a risk factor in cigarette use among adolescents [29, 32]. The significance of industrial promotion on tobacco usage likely represents the effects of tobacco companies’ penetration to LMICs [40]. Similar to other LMICs, the images of tobacco users promoted by tobacco companies, such as success, sociability, sportive, beauty, and feminine liberation [28], might have a stronger impact on adolescents. The study shows that female adolescents who owned tobacco merchandize were significantly more likely to be users of noncigarette tobacco products. For this reason, industry's activities aimed at these adolescents should be of utmost concern to health promoters. Youth tobacco control campaigns that focused attention on the tobacco industry's deceptive activities aimed at youth should be promoted. Such educational campaigns are necessary because the results for school curriculum variables that focused on health effects of tobacco use alone did not show any significant impact in reducing tobacco use.

In this respect, the non-significant association between school curriculum variables and increased tobacco use supports the literature that school programs that only focus on health messages may not work for adolescent populations to reduce the consumption of tobacco products [41]. Therefore school and health educators should be aware that focusing on the health message alone may not be effective to cap smoking habits among school-aged adolescents in the 2 Sudans.

The finding that familial relations increased the likelihood of tobacco use is consistent with evidence from high-income and LMICs [15, 29, 42, 43]. However, the situation presents several policy challenges that should be addressed by the 2 countries including the need to educate parents about SHS, restrict tobacco use and access among young people, and educate adults about the adverse health impacts of smoking on them and their loved ones.

First, the increase in the likelihood of smoking by living in smoking homes and decrease among adolescents who support smoking ban in public arenas calls for the need to educate parents and the general public to have smoke-free environments [29, 44]. This is partly because smoke-free environments including homes and public arenas could help reduce smoking among adolescents [45] and exposure to SHS. There are more than 400 known carcinogens found in SHS [46], and educating smokers and the public about the health implications of smoking and SHS can make smoking socially unacceptable and hopefully reduce tobacco consumption [47]. This needs regulations and legislations that promote smokefree environments, educate adults not to make tobacco products available to youth, and restrict youth access to tobacco products. The fact that knowledge of harmful effects of SHS is associated with decreased tobacco use merits more attention. Knowledge about the negative effects of SHS generally suppresses the propensity to use tobacco among the school-aged adolescents and may reinforce norms that tobacco use is unacceptable.

Second, the fact that cigarette use is socially common and tolerated among adults in the 2 Sudans [15] may increase the perception that cigarette smoking is not harmful. In such circumstances, children may not have the ability to reject tobacco offered to them by adults or tobacco company representatives. With more than 13.3% adolescents who were offered a cigarette product by company representatives (Table 1), the results show that all school-going adolescents in the 2 countries may be susceptible to industry promotion of tobacco products. This implies the need for public health prevention efforts to educate adults about tobacco use and its harmful effects and importance of denying tobacco products to minors.

Third, the fact that tobacco-use behavior of familial relations is significantly associated with adolescent tobacco use in the communitarian societies of the 2 Sudans requires government intervention programs that educate citizens on the adverse health impacts of smoking. There might be need for the government of (north) Sudan to move beyond the ratification to implementation of the FCTC which may lead to creation of laws that protect not only young people from buying cigarette products but also from exposure to SHS. For South Sudan, there is need to hasten and bring forth tobacco control legislations to reduce the excessive use of tobacco in the region and safeguard the future of the next generation from being destroyed by the adverse health impacts of consuming tobacco products.

The study has some limitations worth mentioning. Although the Sudan GYTS data are nationally representative and based on a standardized questionnaire, it is self-reported and there is no independent means of validating the responses from the surveyed adolescents. Additionally, the sample methodology does not account for students who were absent from school on the day of the survey and those who attend no school making it non-representative of regions that may have low school enrolments. In addition, the GYTS data are cross-sectional which limits the temporal analysis of relationship between the dependent and independent variables in the study. Lack of geographical information also limits spatial analyses of tobacco use status in the region. If the spatial information were available it would be possible to analyze and compare regional variations in the prevalence and determinants of tobacco use in the region, especially after the secession of South Sudan from Sudan. Nevertheless, the relatively large representative sample strengthens the inferences and conclusions about tobacco use among school-going adolescents in the 2 countries.

## Conclusion

In conclusion, school programs that focus on health messages alone may not work for the adolescent population. Therefore, legislations that ban all types of tobacco advertisements, promotions, and sponsorships among adolescents are needed in the 2 countries. This study is a major project, to my knowledge, that has explored gender differences in the prevalence and determinants of tobacco consumption among youth (11 -17years) in the 2 Sudans.
